# ADP-Ribosylation Factor Family of Small GTP-Binding Proteins: Their Membrane Recruitment, Activation, Crosstalk and Functions

**DOI:** 10.3389/fcell.2022.813353

**Published:** 2022-02-03

**Authors:** Tiantian Li, Yusong Guo

**Affiliations:** ^1^ Division of Life Science and State Key Laboratory of Molecular Neuroscience, The Hong Kong University of Science and Technology, Kowloon, Hong Kong SAR, China; ^2^ Hong Kong University of Science and Technology, Shenzhen Research Institute, Shenzhen, China; ^3^ Southern Marine Science and Engineering Guangdong Laboratory (Guangzhou), Guangzhou, China

**Keywords:** Arf proteins, cargo sorting, vesicular trafficking, cargo adaptor, Arf GEF, Arf GAP

## Abstract

Members of the ADP-ribosylation factor (ARF) family of guanine-nucleotide binding proteins play critical roles in various cellular processes, especially in regulating the secretory, and endocytic pathways. The fidelity of intracellular vesicular trafficking depends on proper activations and precise subcellular distributions of ARF family proteins regulated by guanine nucleotide exchange factors (GEFs) and GTPase-activating proteins (GAPs). Here we review recent progress in understanding the membrane recruitment, activation, crosstalk, and functions of ARF family proteins.

## Introduction

The small G proteins, refer to the low molecular weight guanine-nucleotide-binding proteins, are responsible for the spatial and temporal regulation of many intracellular processes. These proteins are classified into several major families including Arf, Rab, Ran, Ras, Rad, Rap, and Rho (Liu et al., 2017). Together they form the Ras superfamily. Among those subfamilies, ARF family is further extended to include Arf-related proteins 1 (ARFRP1), Arf-like proteins (ARLs), and SARs, which makes ARF itself a superfamily. The following discussion will focus on ARF family G proteins. The description “ARF family proteins” refers to the whole Arf family, and “Arfs” refers to ARF family proteins Arf1-6 only.

Arfs were first revealed and named as membrane-associated proteins that are important for the ADP-ribosylation of the Gs protein by cholera toxin (Kahn and Gilman, 1986). Gs are the group of G proteins responsible for stimulating the activity of adenylate cyclases (Kahn and Gilman, 1986). Further study revealed that Arfs are GTP-binding proteins and serve as switches to regulate intracellular vesicular trafficking (Gillingham and Munro, 2007). Based on sequence homology, Arfs can be divided to three types: Class I (Arf1-3), II (Arf4-5), and III (Arf6) (Donaldson and Jackson, 2011). Class I Arfs arose early in evolution and are highly conserved, whereas Class II Arfs are less abundant and are absent in some species. Class III only contain one protein, Arf6, which is distinct from Arf1-5 in sequence and biochemical properties (Maranda et al., 2001). In addition, utilizing genome sequencing, a wider range of small G proteins were classified as members in the ARF family, including the Arf-like (Arl) proteins, Sar1, and Arf-related protein 1 (Arfrp1) (Pasqualato et al., 2002; Kahn et al., 2006). These proteins lack ADP-ribosylation activity but share the structural features with Arfs.

Arfs have similar structural organizations, composed of highly conserved effector regions including the switch 1 and switch 2 region, the inter-switch region, and the amphipathic helix at their N terminus (Donaldson and Jackson, 2011). Arfs can switch between a GTP-bound active state and a GDP-bound inactive state, catalyzed by Arf family guanine nucleotide-exchange factors (Arf GEFs) and guanine nucleotide-activating proteins (Arf GAPs), respectively. Binding of the switch domains of Arfs with their corresponding Arf GEFs promotes GTP binding. GTP binding subsequently induces conformational changes to the switch regions of Arfs to mediate membrane recruitment of cytosolic effectors such as cargo adaptors and lipid-modification enzymes (Guo et al., 2014).

The N-terminal amphipathic helix, which is normally myristoylated or acetylated, is essential for the membrane recruitment of ARF family proteins. This feature distinguishes ARF family proteins from other Ras superfamily G proteins. ARF family proteins have specific subcellular localizations. Arf1, Arf4, and Arf5 are shown to predominantly localize to the *cis*-Golgi and Arf3 specifically localizes to the *trans*-Golgi network (TGN) (Donaldson and Jackson, 2011). Arf6 localizes to the plasma membrane and the endocytic system (Donaldson and Jackson, 2011). Arf2 is present in only some of the vertebrates such as mice and rats but not in human (Gillingham and Munro, 2007). Arl5A and Arfrp1 are shown to be TGN-located and Arl14 exhibits plasma membrane and endosomal localizations (Gillingham and Munro, 2007).

## Membrane Recruitment and Activation of ARF Family Proteins

ARF family proteins are recruited to membranes through an N-terminal amphipathic helix which binds to the hydrophobic pocket during GDP-bound state. GTP binding induces a rearrangement of the β-sheet structure in ARF family proteins. During the rearrangement, a loop region called loop λ3 moves away from the core region of GTPase, eliminating the binding site for the N terminus thereby exposing the N-terminal amphipathic helix (Goldberg, 1998). The myristoylation and acetylation of the helix motifs are also important for their membrane associations (Losonczi and Prestegard, 1998; Losonczi et al., 2000; Yang et al., 2020). Upon the exposure of the amphipathic helix, the myristate on the helix of ARF family proteins will be inserted into the lipid bilayer. Through this process, ARF proteins are stabilized onto the membranes.

**FIGURE 1:**
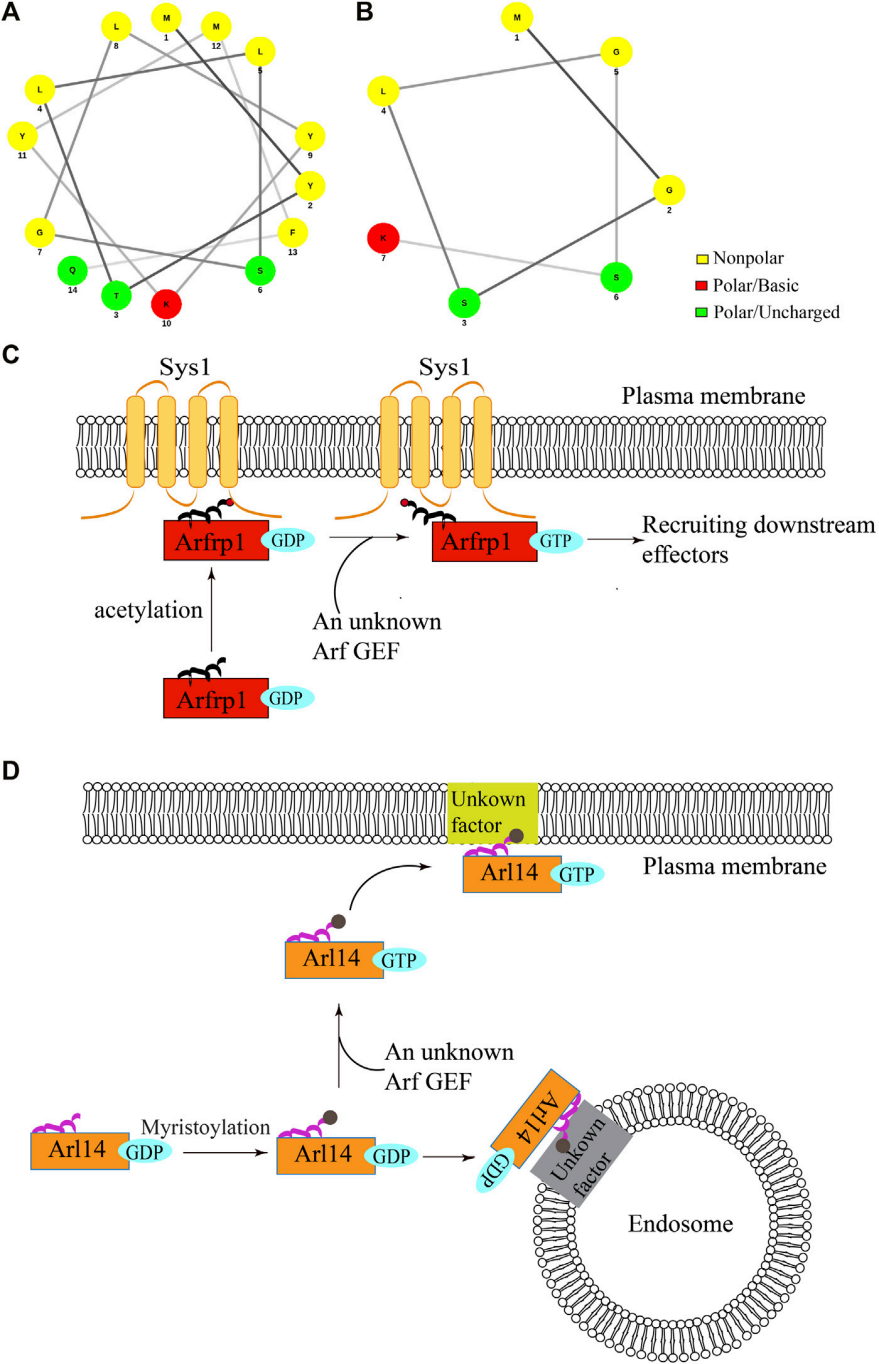
The proposed model of membrane recruitment of Arfrp1 and Arl14. **(A,B)** The helical wheel diagram of N-terminal region of human Arfrp1 **(A)** and Arl14 **(B)**. The diagram was drawn from the following website: http://lbqp.unb.br/NetWheels/. **(C,D)** The proposed model of membrane recruitment of Arfrp1 and Arl14. Upon acetylation at the N-terminal helix, Arfrp1 interacts with the transmembrane protein Sys1 to be recruited to the TGN **(C)**. After myristoylation, GDP-bound Arl14 is preferentially recruited to the endosomes and GTP-bound Arl14 is preferentially recruited to the plasma membranes **(D)**.

The membrane association of ARF family proteins is normally coupled to their GTP-induced activation. Interestingly, a recent study showed that two ARF family proteins, Arfrp1 and Arl14, are recruited to the membranes independent of GTP-binding (Yang et al., 2020) (Figure 1). Arl14 is located at the plasma membrane and endosomes whereas Arfrp1 is located at the Golgi (Yang et al., 2020). The helical wheel diagram suggests that the N-terminal region of Arfrp1 forms an amphipathic helix (Figure 1A) and the N-terminal region of Arl14 forms a short amphipathic helix (Figure 1B) (Gillingham and Munro, 2007). Strikingly, evidence suggests that the N-terminal region of Arl14 and Arfrp1 (amino acids:1-17, referred to as Arfrp1 N-terminal region and Arl14 N-terminal region in the following text) are sufficient for bringing cytosolic proteins to their specific subcellular localizations (Yang et al., 2020). Further analysis indicates that replacing Arl14 N-terminal region with Arfrp1 N-terminal region causes the localization of the chimeric Arl14 protein to switch from the endosome or plasma membrane to the Golgi, which resembles the localization of Arfrp1. Similarly, replacing Arfrp1 N-terminal region with Arl14 N-terminal region causes the localization of the chimeric protein to switch from the Golgi to the plasma membrane. These results suggest that Arfrp1 N-terminal region or Arl14 N-terminal region is sufficient to determine their spatial localization, which may override the spatial determinants provided by the interaction between Arf GEFs and specific ARF family proteins (Yang et al., 2020).

The localizations of Arfrp1 and Arl14 depend on the acetylation and myristoylation at their amphipathic helixes (Yang et al., 2020). Further analysis indicates that a TGN-located transmembrane protein, Sys1, interacts with Arfrp1 N-terminal region and this interaction is critical for the spatial determination mediated by Arfrp1 N-terminal region (Figure 1C) (Yang et al., 2020). Given that the amphipathic helix is partially hidden in the hydrophobic pocket in the absence of GTP binding, we proposed that the hydrophilic surface of the helix interacts with the membrane binding partner of Arfrp1. As Arl14 N-terminus is predicted to contain a short amphipathic helix (Gillingham and Munro, 2007), it is possible that Arl14 amphipathic helix is not tightly associated with the hydrophobic pocket when Arl14 is in an GDP-bound status. Interestingly, the GTP- and GDP-locked forms of Arl14 are preferentially located at the plasma membrane and the endosomes, respectively, (Yang et al., 2020). This analysis suggests that GDP binding directs Arl14 to the endosomes and GTP binding directs Arl14 to the plasma membrane (Figure 1D). The underlying mechanisms that mediate membrane recruitments of Arl14 remain to be further investigated.

Taken together, these analyses reveal a novel mechanism regulating the membrane recruitment of some ARF family proteins, and indicate that GTP-induced activation of some ARF family proteins is uncoupled with their membrane recruitments. This uncoupling pattern is beneficial for ARF family proteins to be recruited to the membrane compartments in the GDP-bound state, thereby increasing the possibility to meet with their specific GEFs. Besides, GTP-independent membrane association of specific ARF family proteins allows the quicker turnover of activated proteins for participating in another round of action. Through this process, the GDP-bound ARF family proteins will remain on the membranes and efficiently participate in the next trafficking cycle. For example, Arfrp1 initiates the sequential recruitment of other ARF family proteins to organelle membranes (Ishida and Bonifacino, 2019). The GTP-independent membrane association of Arfrp1 may accelerate this sequential recruitment process, promoting further GTP-dependent interactions between Arfrp1 and its effectors.

## Mechanistic Insights Into the Functional Roles of Arf GEFs and Arf GAPs

The activity of the ARF family proteins is regulated by Arf GEFs and Arf GAPs. Arf GEFs activate GTPases by promoting the exchange of binding nucleotides from GDP to GTP, whereas Arf GAPs inactivate GTPases by catalyzing the hydrolysis of GTP (Bos et al., 2007; Sztul et al., 2019).

Structural analyses indicate that the catalytic domains of GEFs for different GTPases exhibit distinct structures, and GEFs interact with their substrate GTPases in various ways (Boriack-Sjodin et al., 1998; Goldberg, 1998; Worthylake et al., 2000; Renault et al., 2001; Itzen et al., 2006). But the catalytic domains of GEFs are conserved within a given subfamily (Vetter and Wittinghofer, 2001). Arf GEFs share a conserved Sec7 domain, which is the central catalytic domain (Chardin et al., 1996). GEFs interact with the region between switch 1 and switch 2 of GTPases. During this interaction, the glutamate residues from GEFs are shown to insert into or approach closely to the phosphate-binding loop (P loop) of GTPases, competing with the β-phosphate of the bound GDP to interact with the P-loop lysine (Beraud-Dufour et al., 1998; Thomas et al., 2007).The insertion of glutamic finger caused a rotation of ARF core and the rearrangements in the interswitch region which excludes the N-terminal helix away from the protein core (Renault et al., 2003). Generally, ARF family proteins bind with guanine nucleotide with high affinity (Bourne et al., 1991). The binding of GEFs weakens the affinity between nucleotides and ARF family proteins, thus accelerating the release of nucleotides. The switching-on role of GEFs may attribute to the excess concentration of GTP in the cytoplasm comparing with GDP.

The general role of GAPs is to stimulate the catalytic functions of the GTPases. GAPs and the attacking water molecule approach the GTPases from different angle to catalyze phosphate release (Vetter and Wittinghofer, 2001). Arf GAPs carry a conserved Zn-finger motif and an arginine finger (Cherfils and Zeghouf, 2013). When forming a complex with ARF family proteins, Arf GAPs insert a catalytic residue such as the arginine finger to neutralize the negative charge at the γ-phosphate through forming hydrogen bonds, and this process stabilize the transition state of the GTP hydrolysis (Bos et al., 2007). In addition, a conserved glutamine in the switch 2 region of ARF family proteins is required for GAPs to perform the catalytic role (Cherfils and Zeghouf, 2013). Other than GAPs, previous study has also raised potential binding partners such as coatomer to provide the catalytic arginine finger (Goldberg, 1998). This ARF-GAP interaction subsequently positions the water molecule in an appropriate orientation to perform nucleophilic attack to the β-γ phosphodiester bond thereby promoting GTP hydrolysis (Scheffzek et al., 1997).

## Crosstalk of ARF Family Proteins, Arf GEFs and Arf GAPs

Arf GEFs and Arf GAPs are critical for activation and inactivation of GTPases. Evidence suggests that the activity of Arf GEFs and Arf GAPs are in turn coordinated by their substrates, and ARF family proteins function in a sequential and crosstalk manner rather than act individually. The crosstalk among Rab and ARF family proteins that took place at the Golgi has been reviewed (Thomas and Fromme, 2020). Here, we summarize the crosstalk of ARF family proteins, Arf GEFs, and Arf GAPs.

A study performed in yeast showed that the Golgi-localized Rab proteins Ypt31/32 recruits the GEF for a later acting Rab Sec4, which uncovers a cascade model that early acting Rabs regulate the activation of later acting Rabs through recruiting corresponding GEFs (Ortiz et al., 2002). The activation of Sec4 then promotes the trafficking of vesicles to the sites of exocytosis (Ortiz et al., 2002). Later, the cascade was also shown to be present in the reverse direction, termed GAP cascade, where GAPs were recruited to inactivate early acting GTPases. The GAP cascade was uncovered in yeast where the activation of Ypt32 causes the inactivation of the preceding Rab protein, Ypt1, by recruiting its GAP (Rivera-Molina and Novick, 2009). The biological significance of the GAP cascade is to avoid ectopic activation of GTPases, thereby maintaining the identity of specific organelles. GTPases and their effectors are proposed as markers for specific Golgi cisterna, thus the crosstalk of GTPases and their GEFs and GAPs is critical to the maturation of Golgi (Thomas and Fromme, 2020). More importantly, the GTPases are key regulators of intracellular trafficking process, thus the consecutive GTPase activation mediated by GEFs and GAPs is essential for the precise localization of proteins.

**FIGURE 2:**
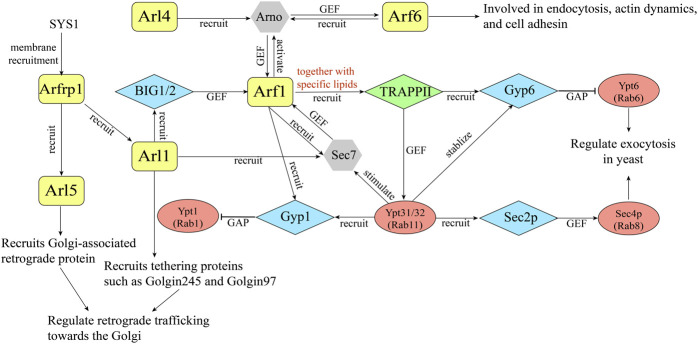
A diagram illustrating an example of GTPase crosstalk between ARF family proteins and Rabs.

Sequential activating cascade is further exemplified in ARF family proteins. In 2003, it was revealed that Arl3p, the yeast homolog of Arfrp1, is required for the TGN recruitment of Arl1p and further recruitment of a Golgin, Imh1p, implying the existence of Arf cascades (Panic et al., 2003; Setty et al., 2003) (Figure 2). Further analyses indicate that in mammalian cells, Arl1 recruits the Arf1 GEF BIG1 and BIG2 to the TGN, thereby activating Arf1 at the trans-Golgi (Christis and Munro, 2012) (Figure 2). Recently, it was reported that Arfrp1 functions to recruit Arl1 and Arl5 to the TGN, presumably through recruiting their specific Arf GEFs in mammalian cells (Ishida and Bonifacino, 2019). At the TGN, Arl5 and Arl1 recruit tethering factors such as Golgins and Golgi-associated retrograde protein (GARP) to mediate the retrograde trafficking from endosome to the TGN (Ishida and Bonifacino, 2019) (Figure 2). The precisely tuned Arl1 localization ensures well-regulated in-and-out vesicle flow at the TGN. These studies suggest that some ARF family proteins mediate the membrane recruitment of specific Arf GEFs, which subsequently regulates the activation of other Arf proteins. The sequential activation of ARF family proteins similar to the Rab cascades allows the spatial and temporal regulation of intracellular trafficking events.

In addition to sequential activation, ARF family proteins are able to recruit their specific Arf GEFs to the membranes which subsequently activate more ARF family proteins, forming a positive feedback loop. The Arf nucleotide-binding site opener (Arno) is a GEF for Arf1 and Arf6. Arno is composed of a coiled-coil region, a Sec7 domain, and a C-terminal PH domain (Stalder and Antonny, 2013). Several studies revealed that the activated form of Arf6 and Arl4 interact with the PH domain of Arno and recruit Arno to the membranes which in turn mediate the activation of Arf1 (Cohen et al., 2007; Li et al., 2007; Hofmann et al., 2007) (Figure 2). Further studies suggested that Arno is preferentially in its autoinhibition form. Upon recruitment to the membrane by the active ARF proteins, the autoinhibition will be relieved to allow Arno to activate ARF family proteins, forming positive feedback activating loop (DiNitto et al., 2007) (Figure 2). Thus, the activity of Arno is mediated by the coordination of autoinhibition and positive feedback.

Beside Arno, another Arf GEF, Sec7, is also shown to be activated by its own product. Sec7 is the yeast ortholog of mammalian BIG1 and BIG2. A recent study indicates that the homology downstream of Sec7 domain (HDS1) of Sec7 interacts with Arf1-GTP to mediate the membrane recruitment of Sec7, initiating the positive feedback loop (Richardson et al., 2012) (Figure 2). Consequently, activated Arf1 stably recruits its activator Sec7 to the membranes. Evidence suggests that the C terminus consisting of HDS1-4 is required for the essential function of Sec7, and the HDS2-4 domains have an autoinhibitory function whereas the HDS1 domain has an activating function (Richardson et al., 2012). In addition to regulate membrane recruitment of Sec7 by interacting with Arf1, Sec7 is proposed to sequester its GEF domain in solution (Stalder and Antonny, 2013). Thus, the HDS1 domain is regarded as a switch, exerting both an inhibitory or activation function. With the broad distribution of Arf1 at the Golgi, Sec7 is proposed to compete with other effectors to bind Arf1-GTP, therefore Sec7 is only activated in the late Golgi/TGN, and this event may serve as a checkpoint for Golgi maturation (Richardson et al., 2012).

**FIGURE 3:**
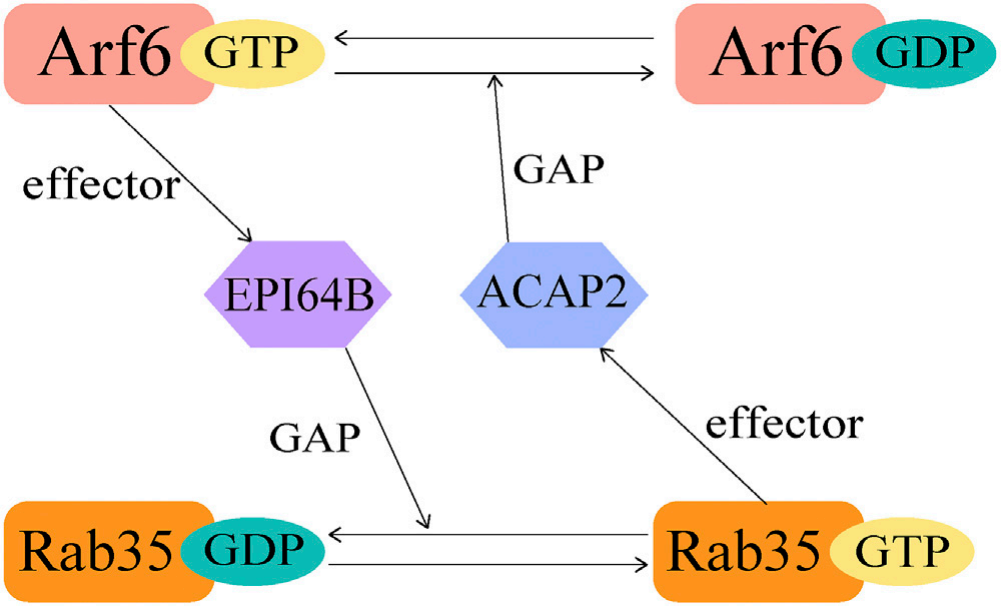
A diagram showing the crosstalk between Rab35 and Arf6.

The crosstalk of GTPases is not limited in Rab-Rab or Arf-Arf, but also happens between Rab and ARF family proteins. It was revealed that Rab35 and Arf6 exhibit a bipartite regulation during activation (Figure 3). In this case, the regulation requires the connecting protein to be both an effector and a GAP/GEF. As an example, Rab35 downregulates Arf6 activity through recruiting ACAP2 during neutrite outgrowth (Kobayashi and Fukuda, 2012). Here ACAP2 functions as the effector of Rab35 and GAP for Arf6. Upon nerve growth factor (NGF) stimulation, Rab35 accumulates at Arf6-positive endosomes and constitutively recruits ACAP2 to inactivate Arf6, together they regulate NGF-induced neurite outgrowth (Kobayashi and Fukuda, 2012). On the other hand, activated Arf6 recruits EPI64B, the effector of Arf6 and GAP for Rab35, thus downregulates the activation of Rab35 at the membrane (Chesneau et al., 2012) (Figure 3). It was previously reported that Rab35 is responsible for the termination of cytokinesis through controlling the fast endocytic recycling pathway, and Arf6 perturbs the endocytic recycling pathway in a similar manner (Kouranti et al., 2006; Chesneau et al., 2012). Thus, this coordinated regulation by Arf6 and Rab35 is essential for these cellular processes.

Another example for GTPase crosstalk is the TRAPPII pathway. It was reported that four GTPases differentially regulate the membrane recruitment and activation of Sec7 Arf-GEF to mediate TGN trafficking. The analyses revealed that Sec7 is an effector of Ypt1 (Rab1), Ypt31/32 (Rab11), Arl1 and Arf1. The membrane localization of Sec7 was primarily affected by Arf1, Arl1, and Ypt1. Subsequently the activity of Sec7 will be significantly stimulated by Ypt31/32 (McDonold and Fromme, 2014). Further studies revealed that Ypt31/32 and its GEF Transport Protein Particle II (TRAPPII) function in a bipartite feedback loop with Arf1 and Sec7 to coordinate vesicle biogenesis (Thomas and Fromme, 2016) (Figure 2). TRAPPII is recruited to the Golgi by mutual efforts of activated Arf1 and anionic lipids. During Golgi maturation, the membranes will be enriched with anionic phospholipids. Then, activated Arf1 recruits TRAPPII to the membrane with the help of anionic lipids to activate Ypt31/32, which in turn stimulates Sec7-mediated Arf1 activation (Figure 2).

Arf1 is also proposed to be the key driver of Rab distribution during Golgi maturation. It was shown that the level of both Ypt1 and Ypt6 decline at the late Golgi before Ypt31/32 reaches the highest activity, suggesting the existence of extra factors to initiate the recruitment of GAP proteins for Ypt1 and Ypt6 (namely the Gyp1 and Gyp6) (Thomas et al., 2021). Arf1 was proposed to be a potential regulator in that both GAPs accumulate right after the peak activation of Sec7 (Thomas et al., 2021). Indeed, Arf1 binds directly to Gyp1 both *in vivo* and *in vitro*, and Gyp1 and Gyp6 were severely mis-localized in Arf1 and TRAPPII mutant (Thomas et al., 2021). Further study revealed that TRAPPII and Arf1 coordinate to recruit Gyp6 and Gyp1, and activate Ypt31/32 whereas inactivate Ypt1 and Ypt6 (Thomas et al., 2021) (Figure 2). Activated Ypt31/32 in turn stabilizes Gyp1 and Gyp6 at the late Golgi membrane (Figure 2).

These bidirectional regulations may represent a general mechanism of the GTPase networks regulating transport pathway. By defining when and where to activate those GTPases through the well-organized recruitment of GEFs and GAPs, the crosstalk ensures that each GTPase is activated precisely at a specific subcellular localization to prevent the overlap of GTPases. The coordination of sequential activation and positive/negative feedback helps concentrate GTPases and their effectors for a better performance *in vivo*. These processes also regulate vesicle biogenesis and balance the incoming and outcoming vesicle flow, thereby ensuring the fidelity of intracellular trafficking process.

## Functional Roles of Arf and Arf-Related Proteins in Vesicular Trafficking

Arf family GTPases are critical mediators of various steps of the vesicle formation processes: the assembly of coat proteins, the sorting of cargo proteins, membrane curvature, and uncoating of vesicles (8).

As aforementioned, GTP-binding of ARF family proteins exposes the amphipathic helixes to insert to the membranes and causes conformational changes of the switch regions to recruit downstream effectors in close proximity to the membranes (Gillingham and Munro, 2007). In addition, evidence suggests that the insertion of the amphipathic helix into the membranes induces membrane curvature and leads to the lipid clustering, and these changes further promote the fission process in trafficking events (Lee et al., 2005; Shih et al., 2011).

Among those Arf and Arf-related proteins in human, Arf1 is the most well-studied and is characterized to regulate membrane recruitment of COPI, APs, GGAs, and the lipid modification enzymes such as Phospholipase D (Donaldson and Jackson, 2011). For example, Arf1 directly interacts with AP-1 in a GTP-dependent manner and this interaction regulate membrane recruitment of AP-1. In addition, binding of Arf1 to AP-1 induces conformational changes in AP-1 (Lee et al., 2008a). AP-1 changes from a closed conformation to an open conformation to allow AP-1 to capture cargo proteins (Crottet et al., 2002; Lee et al., 2008a; Guo et al., 2013; Ren et al., 2013). The interaction between AP-1 and cargo proteins in turn induces the oligomerization of AP-1 and stabilize the interaction between Arf1 and AP-1, contributing to vesicle formation (Lee et al., 2008a; Lee et al., 2008b). AP-1 is also proposed to interact with phospholipids such as PI4P to further stabilize its membrane association (Ren et al., 2013).

Arfrp1 is also proposed to be an activator of AP-1. Evidence suggests that the binding of Arfrp1 to AP-1 opens a non-canonical binding pocket for AP-1 to bind with the tyrosine sorting motif (YYXXF) of its cargo protein, Vangl2, and this process enhances the membrane association of AP-1 (Guo et al., 2013). These analyses indicate that ARF family proteins not only mediate membrane recruitment of cargo adaptors but also regulate the specificity of cargo recognition. Arfrp1 also regulates trafficking of vesicular stomatitis virus G protein (VSVG) and glucose transporters, but the underlying mechanisms remain to be further investigated (Shin et al., 2005; Nishimoto-Morita et al., 2009; Hesse et al., 2012). In addition to mediate cargo sorting, Arfrp1 is shown to indirectly involved in the vesicle tethering process by functioning upstream of Arl1 and Arl5 to recruit Golgi associated retrograde protein (GARP) and golgins such as Golgin-97 and Golgin-245 to the TGN (Ishida and Bonifacino, 2019). As described before, this Arf cascade is critical for the retrograde trafficking towards the TGN.

Similarly, Sar1 recruits the inner COPII component, the Sec23/24 complex to the ER to capture cargo proteins (Lee et al., 2004). In addition, Sar1 was previously reported to directly interact with the dibasic motif on many Golgi-resident glycosyltransferases through its D198 residue to regulate protein export from the ER (Giraudo and Maccioni, 2003; Guo and Linstedt, 2006; Quintero et al., 2010). Recently, it was shown that ER export of a planar cell polarity protein Frizzled6 depends on the direct interaction between the polybasic motif (RRFR) on Frizzled6 and the E62/E63 residues on Sar1A (Tang et al., 2020), suggesting that Sar1 contains multiple cargo binding sites.

Arf1 recruits the lipid transfer proteins ceramide transfer (CERT) and a PI (4) P binding protein, FAPP2, by interacting with their PH domains ^3^. Subsequently, CERT and FAPP2 regulates the transportation of glycolipids and ceramide through their lipid binding domains (Hanada et al., 2003; De Matteis and Godi, 2004). RNAi screening and proteomic analyses indicated that Arf1 associates with its GEF GBF1 and COPI components during lipid droplet formation. These analyses suggest that Arfs regulate lipid transfer and the formation of lipid droplets, which may indirectly affect the membrane trafficking process (Guo et al., 2008).

## Experimental Approaches to Study the Functional Roles of ARF Family Proteins

To better understand how the small GTPases function *in vivo*, it’s critical to develop experimental approaches that are powerful to identify their regulators and effectors. Yeast two-hybrid assay and affinity chromatography are two major approaches to identify the Arf regulators and Arf effectors (Christoforidis et al., 1999; Van Valkenburgh et al., 2001; Fukuda et al., 2008; Gillingham et al., 2014). However, these two approaches are not robust to identify weak and transient protein interactions. New ways have emerged to reveal protein-protein interactions that are weak or transient such as proximity biotinylation (Roux et al., 2012). A method called MitoID is developed by applying this approach in combination with the relocations of proteins to mitochondria to identify binding partners of GTPases (Gillingham et al., 2019). By applying the *in vivo* proximity biotinylation with mitochondrially-localized forms of the GTPases, MitoID can efficiently identify novel interactors including effectors of GTPases (Gillingham et al., 2019).

The vesicle formation assay is another way to identify the Arf regulators or Arf effectors (Huang et al., 2021). During this approach, the vesicle formation assay was performed in the presence or absence of the GTP-locked form of a specific ARF family protein. Vesicles were isolated and the protein profiling of the isolated vesicles were analyzed utilizing quantitative mass spectrometry. This approach can be performed to uncover the cytosolic proteins that are specifically enriched in the vesicle fractions generated in the presence of GTP-locked form of ARF family proteins. A similar vesicle formation approach can be performed in the presence of GDP-locked form of ARF family proteins to identify proteins that specifically interacts with GDP-bound ARF family proteins. This approach has an advantage to identify the Arf binding partners in conditions that lipid bilayer is not disrupted. Using this approach, a novel cytosolic factor, PRRC1, was identified to interact with Sar1A in a GTP-dependent manner on vesicle membranes (Huang et al., 2021).

## Future Perspectives

Although significant progress has been achieved in understanding how ARF family proteins perform their cellular functions, several important aspects remain to be further investigated. What is the spectrum of cargo clients that depend on a specific ARF family proteins to be enriched into transport vesicles? Are the TGN-located ARF family proteins uniformly distributed or localized on specific domains at the TGN? How do those ARF family proteins that are recruited to the membranes independent of GTP released from membranes? What are the functions of ARF family proteins in specialized cells such as immune cells? Future works utilizing advanced tools such as the liposomal binding assay, the MitoID, and vesicle formation assay will provide insights into these important aspects. Super-resolution imaging analysis and live imaging approaches will shed light on the vesicular trafficking process mediated by ARF family proteins. It is also critical to study the connection between Arf-mediated signaling and other intracellular signaling pathways.
